# Discriminating nucleosomes containing histone H2A.Z or H2A based on genetic and epigenetic information

**DOI:** 10.1186/1471-2199-10-18

**Published:** 2009-03-04

**Authors:** Alain L Gervais, Luc Gaudreau

**Affiliations:** 1 Département de Biologie, Université de Sherbrooke, Sherbrooke, Québec, Canada

## Abstract

**Background:**

Nucleosomes are nucleoproteic complexes, formed of eight histone molecules and DNA, and they are responsible for the compaction of the eukaryotic genome. Their presence on DNA influences many cellular processes, such as transcription, DNA replication, and DNA repair. The evolutionarily conserved histone variant H2A.Z alters nucleosome stability and is highly enriched at gene promoters. Its localization to specific genomic loci in human cells is presumed to depend either on the underlying DNA sequence or on a certain epigenetic modification pattern.

**Results:**

We analyzed the differences in histone post-translational modifications and DNA sequences near nucleosomes that do or do not contain H2A.Z. We show that both the epigenetic context and underlying sequences can be used to classify nucleosomal regions, with highly significant accuracy, as likely to either contain H2A.Z or canonical histone H2A. Furthermore, our models accurately recapitulate the observed nucleosome occupancy near the transcriptional start sites of human promoters.

**Conclusion:**

We conclude that both genetic and epigenetic features are likely to participate in targeting H2A.Z to distinct chromatin loci.

## Background

Nucleosome core particles are protein complexes highly conserved in all eukaryotes, present at every 160 to 240 DNA base pairs [1] and composed of eight histone molecules (two of each H2A, H2B, H3 and H4). They are primarily responsible for compaction of the eukaryotic genome and also play a role in various cellular processes, including transcriptional gene regulation, DNA replication, and DNA repair. Eukaryotic cells have evolved to use histone variants that differ from canonical histones, and which can define specialized areas of chromatin. While canonical histones are largely expressed during DNA replication, histone variants are usually expressed throughout the cell cycle. The intensively studied histone variant H2A.Z is highly conserved in evolution, even more so than H2A [2].

The first evidence of a transcriptional involvement for H2A.Z came from experiments in *Tetrahymena thermophila*, where H2A.Z was found to reside exclusively in the transcriptionally active macronucleus [3]. Later experiments in yeast have shown that H2A.Z can directly affect transcription [4,5]. In yeast, H2A.Z was shown to prevent the spread of heterochromatin into euchromatin [6]. We and others have found that H2A.Z is preferentially localized to the initiator region of many yeast genes, where two H2A.Z-containing nucleosomes flank a nucleosome-free region [7-10]. Importantly, the presence of H2A.Z at promoters has been shown to correlate positively with transcriptional activity in human cells [11], a situation that appears to be in contrast to what has been documented in yeast, where there is an inverse correlation with transcriptional activity [7,8,10]. Nonetheless, there is one report in yeast that does not find any correlation of H2A.Z presence with transcription rate [9]. The authors of this study used arrays covering a single yeast chromosome, and we believe that they may have missed some highly transcribed genes, precluding them from identifying this inverse correlation. In addition to transcriptional regulation, H2A.Z is critical for other functions, such as proper development [12], centromere structure [13], and genome integrity [14,15]. There is evidence that H2A.Z itself is not a self-perpetuating epigenetic mark [16]. It is therefore conceivable that protein complexes able to deposit H2A.Z on chromatin are targeted to specific loci by recognizing a DNA sequence pattern or a combination of epigenetic marks. A complex containing Swr1, a Swi/Snf-related ATPase, is able to catalyse deposition of H2A.Z in yeast [17-19]. In mammals, two complexes have been identified that contain orthologs of Swr1: SRCAP and p400. Both complexes are known to deposit H2A.Z within chromatin both *in vitro *and *in vivo *[20-22]. The mechanism by which they target H2A.Z to specific DNA loci is not clear. In yeast, inserting a short DNA fragment containing a sequence similar to the consensus Reb1 recognition site followed by a polyT tract is sufficient to target the formation of a nucleosome-free region flanked by two nucleosomes containing H2A.Z, in a region previously shown not to contain H2A.Z [9]. In addition, the Tup1 corepressor has been shown to cooperate with the SWR1 complex in H2A.Z deposition both at the GAL1 and SUC2 promoters in yeast [23]. Although it is known that Tup1 interacts with hypoacetylated H3 and H4 histone tails [24], the exact mechanism by which it is targeted to the GAL1 promoter still remains unsolved [23]. In *Caenorhabditis elegans*, the PHA-4 transcription factor (which belongs to the FoxA family) binds a known DNA sequence and recruits H2A.Z to the promoters of genes involved in pharyngeal development [25]. However, a sequence pattern that is able to promote the deposition of H2A.Z has yet to be found in mammalian cells. Intriguingly, however, we have been able to show that H2A.Z can localize to p53 binding sites at general target genes tested, which could suggest a role for transcriptional activators in targeting H2A.Z to certain loci [22]. Another mechanism by which a deposition complex may be targeted to chromatin loci is by recognizing histone post-translational modifications, which extends the information that may be interpreted from chromatin, thus forming a "histone code" [26]. Proteins containing bromodomains and chromodomains are able to recognize acetylated and methylated histone residues, respectively. Importantly, Swr1 co-purifies with Bdf1 (Bromodomain Factor 1), a protein that recognizes acetylated H3 and H4 histone tails [18]. The p400 complex contains Brd8 (Bromodomain containing 8), an ortholog of Bdf1, which could have the same role. Taken together, these observations motivated us to search both for a short DNA motif and an epigenetic pattern that may be responsible for promoting H2A.Z deposition to chromatin.

It has long been known that DNA sequences differ in their binding affinity to histone octamers [27], up to a thousand-fold [28], which has led to the search for a pattern able to predict nucleosome binding affinity. A Fourier analysis of 177 nucleosome sequences from the chicken genome revealed a clear periodicity signal for AT dinucleotide pairs, which is repeated with a period of 10.2 base pairs [29]. This period corresponds roughly to a turn of the DNA double helix. Both natural and artificial sequences selected for high nucleosome affinity show a similar periodic pattern [28,30]. Dinucleotide base pairs differ in structural properties such as bendability and twistability [31]. Indeed, the number and phase of AT nucleotide pairs influences sequence affinity to nucleosomes [32]. This is the rationale for the "flexibility model", which will be described below. The crystal structure of H2A.Z is remarkably similar to that of H2A despite a low sequence similarity of 60% [33,34]. Two regions stand out from this structure: the docking domain, which creates an interaction surface with the H3–H4 tetramer, and the histone fold, which is a conserved motif common to core histones. The differences present in the docking domain and histone fold regions lead to changes between H2A.Z-H2B dimers and in their interaction with H3–H4 tetramers, as compared to H2A-H2B [33]. Furthermore, nucleosomes containing H2A.Z display increased mobility and decreased correlations between internal motions, particularly in the L2 loop, which closely interacts with DNA [35]. Taken together, these observations motivated us to explore the possibility that longer DNA sequence features may facilitate H2A.Z deposition within chromatin.

It should be noted that H2A.Z itself is the target of post-translational modifications, such as acetylation, in yeast [36,37] and ubiquitilation in human cells [38]. In yeast, H2A.Z can be acetylated at four lysines, K14 being the most abundantly modified one. Acetylation of H2A.Z is associated with gene activity [36] and is required for the maintenance of NuA4-dependant telomeric heteochromatin boundaries [37]. Ubiquitylation marks H2A.Z within facultative heterochromatin, such as the inactive X chromosome of female cells [38]. The genome-wide presence of variant histone H2A.Z has been studied using next-generation sequencing in three organisms as of yet: yeast [39], drosophila [40] and humans [11]. Recently, high-resolution localization data has been published for 37 histone post-translational modifications present on human nucleosomes [11,41], allowing for a systematic search of a pattern that could shed some light on H2A.Z localization preferences. Our analysis uses these multiple sources of information to identify features that differentiate H2A.Z from H2A loci in humans, which distinguishes it from earlier studies. Here we show that models based on both genetic or epigenetic information are able to predict the H2A.Z or H2A status of nucleosomes with an accuracy significantly greater than random, and that a flexibility-driven model of those sequences can predict the distribution of H2A.Z-containing nucleosomes observed near transcriptional start sites *in vivo*.

## Methods

We sought to identify either a genetic or an epigenetic signature discriminating between H2A.Z- or H2A-containing nucleosomes. In order to achieve this, we used data published from the studies of Barski *et al*. and Wang *et al*. (see Table 1), which rely on high-throughput sequencing of immunoprecipitated DNA fragments ("ChIP-Seq"). There are over seven million such coordinates made available using H2A.Z and H2A antibodies, and between two million and sixteen million for all other post-translational modifications surveyed. Coordinates referring to genomic regions where both H2A.Z- and H2A-containing nucleosomes were identified, which we call "contradictions", were considered to be H2A.Z. The rationale behind this is H2A.Z replaces H2A in nucleosomes following replication and the observation of H2A.Z in at least some cells in a population indicates that a nearby pattern promoting H2A.Z deposition could exist. This assumes that deposition of S-phase H2A is largely untargeted and that the deposition of replication-independent H2A.Z is targeted. Removal of contradictions left more than four million coordinates in the H2A dataset.

**Table 1:** Summary of the datasets used in this study

**Source**	**Contents**
Barski *et al*. [11]	H2A.Z, H2A-H4R3me2 and 19 histone methylations (H2BK5me1, H3K27me1, H3K27me2, H3K27me3, H3K36me1, H3K36me3, H3K4me1, H3K4me2, H3K4me3, H3K79me1, H3K79me2, H3K79me3, H3K9me1, H3K9me2, H3K9me3, H3R2me1, H3R2me2, H4K20me1, H4K20me3)

Wang *et al*. [41]	18 histone acetylations (H2AK5ac, H2AK9ac, H2BK120ac, H2BK12ac, H2BK20ac, H2BK5ac, H3K14ac, H3K18ac, H3K23ac, H3K27ac, H3K36ac, H3K4ac, H3K9ac, H4K12ac, H4K16ac, H4K5ac, H4K8ac, H4K91ac)

Throughout this paper, classifiers are trained or tested on datasets having the same number of H2A.Z and H2A entries, which implies that the expected accuracy of a random classifier operating on these datasets is 50%. Entries are selected randomly from the larger dataset to equal the size of the smaller dataset. All classifiers are tested on entries distinct from the training datasets, and reported performances correspond to performance evaluated on the test datasets. When referring to random datasets, we refer to datasets used as negative controls where H2A.Z and H2A entries have been intermixed, and we do not expect to find a pattern discriminating such datasets.

### Epigenetic information

In an effort to identify an epigenetic pattern possibly facilitating the deposition of the H2A.Z in chromatin, we used the genomic coordinates of H2A.Z- and H2A-containing nucleosomes, and of all 37 post-translational modifications. For each genomic region, we verified if it co-localized with any of the post-translational modifications. Co-localization was defined as a distance between centers of nucleosomes inferior than 300 base pairs, which allows identification of histone post-translational modifications occurring near the coordinates of that nucleosome or near those of its immediate neighbour. This provided us with information on many post-translational modifications seen within the vicinity of genomic locations where H2A.Z- and H2A-containing nucleosomes were found.

To identify which post-translational modifications better discriminated between the two datasets, we used the C4.5 algorithm [42], which builds a decision tree by measuring the information gained by splitting the data using each modification. The tree is built iteratively until no post-translational modifications remain or certain termination conditions are met. The inferred decision tree can be interpreted and later be used for classification.

### Genetic sequence information

The genomic sequences associated to H2A.Z- or H2A-containing nucleosomes were extracted by extending the aforementioned short read coordinates to the full length of DNA around a nucleosome (146 base pairs), taking into account the strand of the short read, which is necessary to appropriately identify the nucleosome center position [43]. Those coordinates were further extended, centered on the nucleosome, to either 150 or 300 base pairs. Sequence datasets were generated by extracting the sequences at these coordinates from the UCSC human genome, version 18 [44].

We have attempted to associate a pattern to H2A.Z sequences using multiple methods, which are detailed in the following subsections. These include using traditional motif finding algorithms, an exhaustive word counting analysis, a blended-spectrum support vector machine search and various Markov models, of which our flexibility model is a subtype.

#### Motif finding algorithms

In an attempt to identify a short DNA sequence motif to the deposition of H2A.Z in nucleosomes, we extracted the 100 most abundant, non-repeated H2A.Z sequences that were within one thousand base pairs from transcriptional start sites. We then searched for over-represented sequences using the BioProspector [45] and MEME [46] motif finding algorithms. To assess the validity of a putative motif found by these algorithms, we extracted the corresponding weight matrix from the algorithm output, searched for highest-scoring matches genome-wide, and evaluated if a co-localization could be found with H2A.Z nucleosome coordinates.

#### Word counting

Recognizing that the practical restrictions on the number of sequences that could be used in many traditional motif finding algorithms might preclude us from finding motifs enriched in the whole H2A.Z datasets, we used a simple word counting technique that would be able to take advantage of all the sequences in the datasets. A word is a subsequence of smaller length in a nucleosomal sequence. We computed the observed frequency of all possible words of length 1 to 12 in all datasets and computed their enrichment in H2A.Z datasets as compared to H2A datasets.

#### SVM feature search

Support vector machines have been applied successfully to identify nucleosome-forming sequences [47]. We have applied a similar technique to discriminate H2A.Z from H2A sequences, using the software package GIST [48] trained on a feature space composed of observed frequencies of all words of lengths 1 to 6 of all sequences in both datasets. For practical reasons, namely memory requirements, we had to limit training to the 2000 most frequently observed sequences of each dataset.

#### Markov models

The use of nucleosome coordinates that have essentially a single base pair resolution allowed us to build positional sequence model of similar resolution. We sought to model H2A.Z and H2A nucleosome sequences using positional Markov models of variable order, and a positional flexibility model similar to an order 1 Markov model where the probabilities of observing a flexible dinucleotide pair (AT/AA/TT/TA) is evaluated against all other dinucleotide pairs. The models are positional in the sense that model parameters are computed, and therefore different, for each sequence position. Non-positional Markov models, in which model parameters are the same for the whole sequence, have also been trained and evaluated as a basis for comparison.

A formal description of the positional Markov model used is given here, and to our knowledge such models have not been described elsewhere, at least in the context of biological sequences. Non-positional Markov models of varying order, when applied to nucleotide sequences, are used to evaluate the probability of observing a nucleotide preceded by a varying number of other nucleotides that depend on this order. For example, a third-order Markov model can be used to evaluate the probability *P *(*A*|*T T T*), which translates to the probability of observing an adenine preceded by three thymines in some biological sequence. We extend this model considering the position at which those nucleotides are observed as a parameter of model: *P *(*A*|*T T T*, *j *= 10), which translates to the probatility of ovserving an adenine preceded by three thymines *at position 10 *of the sequences. Given a list **S **of *n *sequences of equal length *l*, the foreground probability *f*_*i*, *j *_of observing the short nucleotide sequence mapping to integer *j *at position *i *of the sequences is based on equation 1. To simplify description of the method, one should assume that function *β*(**s**, *i*, *o*) maps the short subsequence in **s **represented by nucleotides {*s*_*i*-*o*_, *s*_*i*-*o*+1_,..., *s*_*i*_} to a unique integer value, where **s **∈ **S**. Function *β*(**s**, *i*, *o*) is trivially implemented knowing that there are 4^*o*+1 ^possible indices for nucleotide sequences of length *o *+ 1.

(1) $$\begin{array}{ccc} f_{i,j}= & \frac{\sum_{\forall s\in S}\alpha (j,\beta (s,i,o))}{n} & \begin{array}{l} \forall i\text{ where }o\leq i\leq l\\ \forall j\text{ where }0\leq j<4^{o+1}\\ o=\text{order of the model} \end{array} \end{array}$$

where

(2) $$\begin{array}{c} \alpha (j_{1},j_{2})= \end{array}\{\begin{array}{cc} 1 & \text{if}j_{1}=j_{2}\\ 0 & \text{otherwise} \end{array}$$

Similarly, the background probability *b*_*j *_of observing the short nucleotide sequence mapping to integer *j *is based on equation 3. Notice that all positions are treated equally.

(3) $$\begin{array}{ccc} b_{j}= & \frac{\sum_{\forall s\in S}\sum_{i=o}^{l}\alpha (j,\beta (s,i,o))}{n(l-o)} & \begin{array}{c} \forall j\text{ where}0\leq j<4^{o+1}\\ o=\text{order of the model} \end{array} \end{array}$$

Alternatively, the parameters *b*_*j *_can be computed from the whole genome sequence rather than from the input sequences **S**. The scoring function uses the parameters computed to evaluate the log-odds of observing a particular sequence, as is detailed in equation 4.

(4) $$\text{Score}(s)=\sum_{i=o}^{l}\log_{2}\frac{f_{i,\beta (s,i,o)}}{b_{\beta (s,i,o)}}$$

The parameters *f*_*i*, *j *_and *b*_*j *_are computed separately on the H2A.Z and H2A sequences. When presented with an unknown sequence, the classifier assigns it to the most likely class according to this score.

## Results and discussion

### Epigenetic information can be used to predict H2A.Z localization

**Figure 1:**
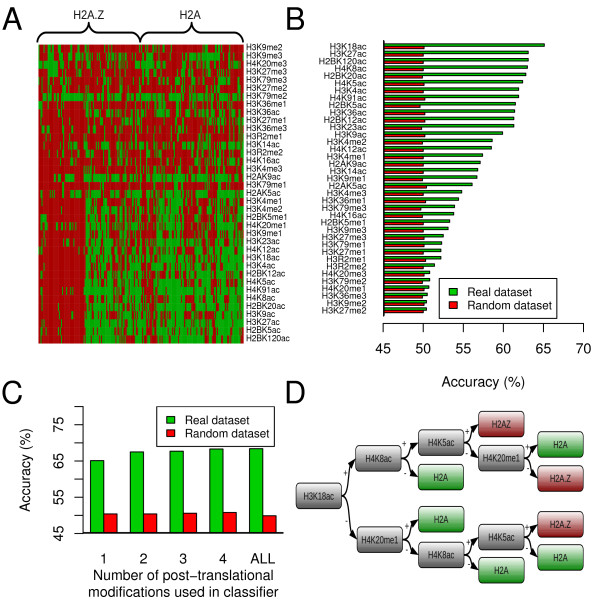
**Epigenenetic information can be used to predict if a nucleosome is likely to contain H2A.Z**. **A**. Histone post-translational modifications neighbouring randomly selected genomic regions where a H2A.Z- or H2A-containing nucleosome was found. Red indicates presence of a modification and green indicates absence. **B**. Accuracies of classifiers trained on a single post-translational modification using the C4.5 algorithm. Post-translational modifications vary greatly in their potential to predict the H2A.Z status of a nucleosome. Most of the best modifications are acetylations. **C**. Accuracies of the best classifier trained on a combination of the specified number of post-translational modifications. Using multiple post-translational modifications improves the overall classification accuracy. **D**. Best decision tree inferred using the C4.5 algorithm using four post-translational modifications. Three modifications in this tree (H3K18ac, H4K5ac and H4K8ac), if present in a particular genomic region, guide the classification toward H2A.Z, while the other (H4K20me1) guides it toward H2A.

We first examined if epigenetic information such as histone acetylations and methylations may be used to predict the presence of H2A.Z-containing nucleosomes observed *in vivo*. As previously stated, we used the coordinates published by Barski *et al*. and Wang *et al*. to identify which post-translational modifications were found in proximity to each H2A.Z and H2A-containing nucleosomes. We chose to model post-translational modifications as either present or absent, as represented in Figure 1A. The figure shows a clustered view of one hundred entries of H2A.Z and H2A locations that have been selected randomly from the millions of entries available. The presence of a modification is determined according to the previously mentioned co-localization criteria (see Methods).

If we restrict the C4.5 algorithm to consider any single post-translational modification, we can show that not all post-translational modifications are equal in their ability to predict the H2A.Z status of a nucleosome (Figure 1B), ranging from having highly significant success (65.1% accuracy for H3K18ac) to not performing better than a random classifier (50.4% accuracy for H3K27me2). Note that the top thirteen post-translational modifications predictive of H2A.Z presence are acetylations. We speculate that this might be reflective of bromodomains in proteins of the deposition complexes.

In an effort to identify which combinations of post-translational modifications best predict the presence of H2A.Z, we tested each possible combination of up to four modifications. We limited the number of training and testing entries to 50,000 randomly chosen regions, keeping running time within reasonable limits while still achieving statistically highly significant results. Predictably, there is an increase in classifier accuracy with an increasing number of post-translational modifications used in training (Figure 1C). The best result was obtained considering H3K18ac, H4K5ac, H4K8ac and H4K20me1 (accuracy of 68.3%, p-value < 1.0 × 10^-324^, binomial test). The best classification tree using four post-translational modifications inferred by the C4.5 algorithm is given in Figure 1D. Post-translational modifications H3K18ac, H4K5ac and H4K8ac are positive predictors of H2A.Z presence, while H4K20me1 is a negative one. The identified post-translational modifications are not guaranteed to occur on the same nucleosome, since the chromatin immuno-precipitation experiments from which the datasets used are derived are distinct and separate experiments, and also because the distance threshold to identify local post-translational modifications was sufficient to span one nucleosome on each side.

We conclude from these results that it is indeed possible to use epigenetic information to predict if a locus is more likely to be enriched in H2A.Z relative to H2A. H2A.Z binding has been previously strongly associated with H3K4me2 [11], and importantly we have also been able to score this as the prevalent histone methylation mark associated with H2A.Z.

### Genetic information can also be used to predict H2A.Z localization

**Figure 2:**
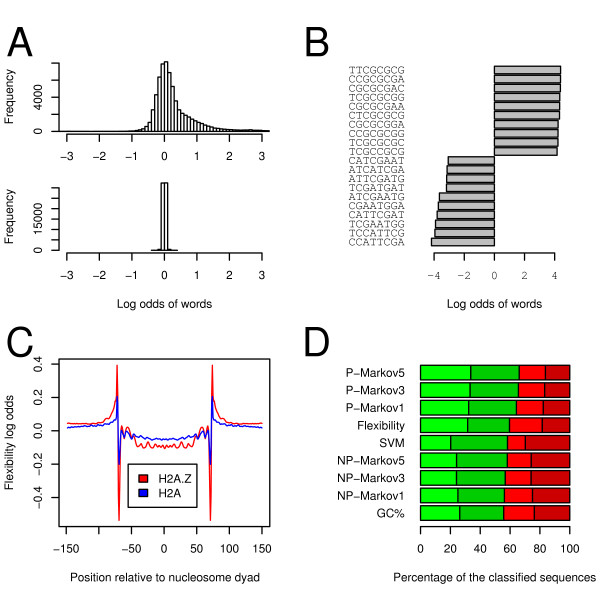
**Genetic information can also be used to predict if a nucleosome is likely to contain H2A.Z**. **A**. Top panel is a histogram of the log-odds of all words of eight base pairs in the H2A.Z dataset compared with the H2A dataset. Bottom panel shows the same analysis carried out on the randomized dataset. **B**. Log odds of the most enriched and most depleted words in the H2A.Z versus the H2A dataset. **C**. Flexibility profile of H2A.Z- and H2A-containing nucleosome sequences. These curves represent the positional flexible dinucleotide log-odds of the flexibility models described, trained on all H2A.Z and H2A sequences including their reverse complement, using a background probability calculated on input sequences without regard to position. H2A.Z-associated sequences are slightly more rigid than their H2A counterparts. **D**. Classification results for some of the sequence-based classifiers investigated. True positives in light green, true negatives in dark green, false positives in light red and false negatives in dark red. GC%: A model based solely on GC content of the sequences. MarkovX: A model based on a positional Markov model of order X (see text). NPMarkovX: A model based on a non-positional Markov model of order X. Flexibility: A model based on dinucleotide flexibility (see text). SVM: A model based on a support vector machine.

Several high-scoring motifs have been found using the MEME and BioProspector algorithms. However, we were not able to find co-localization of genome-wide instances of any motif and H2A.Z (data not shown). The word-counting analysis revealed that some words are enriched in H2A.Z datasets relative to the H2A dataset (Figure 2A). In fact, there are more enriched words than depleted words (asymmetry coefficient of 0.8). This is not the case for words in the random dataset. The most enriched and depleted words formed of eight base pairs are given in Figure 2B. We present computed results using this word length because it is the longest length tested for which the odds computed did not suffer from the small number of corresponding sequences. Indeed, the longer the sequence considered, the less likely it is to occur in the datasets. These results indicate that although we were unable to find a small DNA sequence associated with H2A.Z, some words are clearly enriched in H2A.Z sequences.

To investigate if longer sequence models could explain H2A.Z distribution, sequences from the H2A.Z and H2A datasets were modelled using the positional flexibility model described earlier. We have observed a pattern that is different for H2A.Z and H2A-associated sequences (Figure 2C), suggesting a possible sequence bias. We then tested the different sequence models mentioned earlier and evaluated their performance (Figure 2D). The flexibility model achieves an accuracy of 59% over all sequences tested. This classification can be improved significantly using a fifth-order positional Markov model, which achieves an accuracy of 66%. However, using even higher order Markov models does not improve accuracy (data not shown). Notice that although the most enriched words are GC-rich, and the flexibility model indicates that H2A.Z sequences are more rigid, a model based solely on the percentage of GC base pairs in the sequences has the lowest accuracy of the models tested (Figure 2D). Different measurement methods of the stability of nucleosomes containing H2A.Z produce conflicting results [49,50], which could be resolved by the fact that nucleosome stability is affected by acetylation [51]. We find that nucleosomes containing H2A.Z are present at sequences more rigid than H2A, which we speculate could be cause by its increased stability [51]. We have thus shown that the DNA sequence compacted by a nucleosome contains features that can be used to predict if it is more likely to be harboring H2A.Z or H2A.

### The flexibility model is sufficient to predict the presence of H2A.Z bordering transcriptional start sites

**Figure 3:**
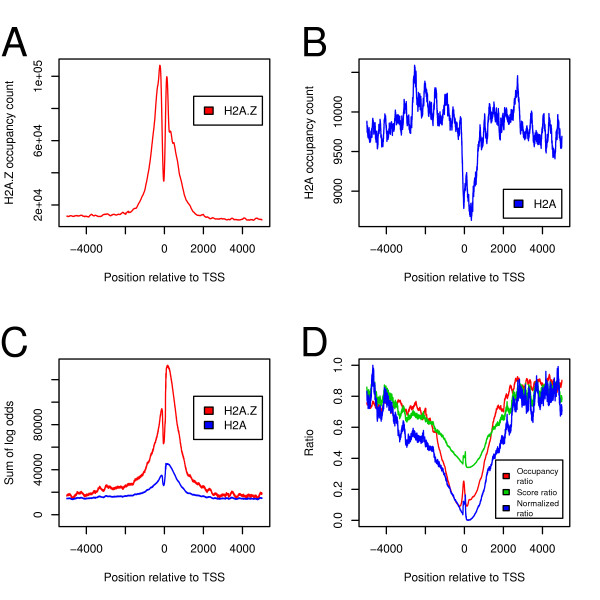
**The flexibility model can recapitulate the H2A.Z presence pattern bordering transcriptional start sites observed in vivo**. **A**. H2A.Z occupancy calculated from data originating from Barski *et al*. Occupancy is calculated by counting how many times a nucleosome is found on each base pair. **B**. H2A occupancy, calculated as in panel A. **C**. Scores of the H2A.Z and H2A flexibility models over all human transcriptional start sites. Those regions are clearly a better fit for the H2A.Z model. **D**. Model and occupancy ratios. The occupancy ratio curve is calculated by dividing the occupancy counts of the H2A dataset by that of the H2A.Z dataset. The score ratio is calculated similarly by using the scores in panel C. The normalized curve is calculated by scaling the values of the score ratio curve between 0 and 1. The occupancy ratio curve was not normalized in any way.

We next asked if the models computed in the previous section could be validated by observations made *in vivo*. Because H2A.Z-containing nucleosomes are enriched near transcriptional start sites, we chose to focus our analysis to these regions. Figures 3A and 3B show the nucleosome occupancy counts observed *in vivo*, computed by incrementing a vector of counters for all base pairs covered by all observed nucleosomes. In contrast to the datasets used to build our sequence models, we did not filter contradictions (regions where both H2A.Z and H2A were present) when computing occupancy. An enrichment of H2A.Z nucleosomes near, but not directly at, transcriptional start sites can be observed (Figure 3A). Also, reduced H2A occupancy near transcriptional start sites has been found (Figure 3B).

We applied the flexibility sequence model to predict H2A.Z and H2A nucleosome occupancy near all human transcriptional start sites in the UCSC genome database. If one considers that the predicted affinity of a sequence to nucleosomes is equal to the score of either model, we can clearly see regions of high predicted nucleosome forming potential at both sides of transcriptional start sites, separated by a region of low nucleosome-forming potential (Figure 3C). Predicted H2A.Z nucleosome-forming potential is higher near transcriptional start sites, which agrees with previously reported genome-wide nucleosome occupancy assays [11]. Furthermore, a short region of low nucleosome-forming potential between the two H2A.Z peaks is predicted, which is directly aligned with the H2A.Z depletion observed in Figure 1A. This is reminiscent of the nucleosome-free region present at yeast gene promoters [7-10,52]. We then chose to compute the observed H2A to H2A.Z occupancy ratio, as well as the predicted H2A to H2A.Z score ratio (Figure 3D). These ratios give surprisingly similar results, and the two curves have a Spearman's correlation value of 0.90. With these results, we find that the flexibility model used accurately reflects the observed H2A to H2A.Z occupancy ratio near transcriptional start sites observed *in vivo*.

## Conclusion

The purpose of this paper was to identify either a sequence pattern or an epigenetic pattern that could account for H2A.Z localization within specific genomic loci. Our data show that it is possible to predict whether a nucleosome is more likely to contain H2A.Z or canonical histone H2A using both genetic or epigenetic information with highly significant accuracy, and that the flexibility model was able to recapitulate the observed pattern of H2A.Z deposition observed *in vivo*. Histone modifications H3K18ac, H4K5ac and H4K8ac are positively associated with H2A.Z, while H4K20me1 is negatively associated. The DNA flexibility model suggests that H2A.Z is more likely than H2A to be found near transcriptional start sites. Since models using both genetic and epigenetic information achieve similarly good accuracy, we speculate that mechanisms recognizing histone post-translational modifications and influenced by DNA sequence are likely to exist *in vivo*.

The classifiers we tested were trained on data originating from all genomic regions, irrespective of transcriptional activity or neighboring genomic features such as coding regions or telomeres. They provide a birds-eye view of the features that are most likely to influence H2A.Z deposition genome-wide, and may help prioritize further biological experiments. It is possible that the best predictors of deposition for subsets of H2A.Z molecules (near telomeres, for example) are different than those observed genome-wide.

## Availability

The source code written for this study can be downloaded from .

## Authors' contributions

AG and LG designed the research. AG wrote the code and did the experiments. AG and LG wrote the paper. All authors read and approved the final manuscript.
